# Identification coronavirus (SARS-CoV-2) and physicochemical qualities in various water sources and the efficiency of water treatment plants in their removal- case study: Northwest region of Iran

**DOI:** 10.1007/s13201-022-01615-5

**Published:** 2022-04-04

**Authors:** Farhad Jeddi, Chiman Karami, Farhad Pourfarzi, Abdollah Dargahi, Mehdi Vosoughi, Ali Normohammadi, Anoshirvan sedigh, Morteza Alighadri, Hadi Sadeghi

**Affiliations:** 1 Department of Genetics and Pathology, School of Medicine, Ardabil University of Medical Sciences, Ardabil, Iran; 2 Department of Microbiology, Parasitology and Immunology, School of Medicine, Ardabil University of Medical Sciences, Ardabil, Iran; 3 Digestive Disease Research Center, Ardabil University of Medical Sciences, Ardabil, Iran; 4 Social Determinants of Health Research Center, Ardabil University of Medical Sciences, Ardabil, Iran; 5 Department of Environmental Health Engineering, School of Health, Ardabil University of Medical Sciences, Ardabil, Iran

**Keywords:** SARS-CoV-2, Physicochemical qualities, Water sources, Monitoring, Water treatment plants

## Abstract

The presence of SARS-CoV-2 virus in water resources and the transmission of diseases caused by it is one of the factors threatening the quality of water resources. This study for the first time concentrates on the presence of SARS-CoV-2 in water resources an urban location. In the present study, the samples were collected from known depth (30–50 cm) of rivers, dams and lakes. In each sample of water collected, different parameters such as residual chlorine, pH (phenol red), turbidity, total dissolved solids and temperature were also measured. Out of 267 samples, two samples were detected to be positive which their Ct values were 34.2 and 35.67. The existence of viable form of this virus in water and wastewater may be associated with issues for providing public health and difficulties in implementation of pandemic control strategies, and this situation can be exacerbated in developing countries that do not have adequate access to sanitation and safe water.

## Introduction

The presence of viruses in water resources and the transmission of diseases caused by it is one of the factors threatening the quality of water resources (Mancuso et al. 2021). With the outbreak of coronavirus in 2019 in the world and its identification in municipal wastewater, the possibility of the presence of this virus in water sources and services is raised (García-Ávila et al. 2020). Coronaviruses are RNA-positive viruses and belong to the coronavirus family and the order Nidovirales. These viruses are widespread in human and mammalian species (Karami et al. 2021a, b). The severe acute respiratory syndrome (SARS) and Middle East respiratory syndrome (MERS) are the disease, which has been two large epidemics due to coronaviruses (Dargahi et al. 2021a; Dargahi et al. 2021b). At the end of 2019, another disease, which has been become pandemic a novel mutation of the coronavirus (categorized as SARS-CoV-2), was recognized and rapidly spread in the world (Lahrich et al. 2021; Vosoughi et al. 2021). According to reports, the number of infected people with this new mutation, till June 2021, was more than 180.4 million individuals affected, among which more than 3.9 million people have passed away [https://www.worldometers.info/coronavirus/]. This disease has been associated with many huge disasters so that control of this infection has not been possible in the even with the best healthcare systems (Langone et al. 2021). The economic damage of trillions of dollars and the unclear future have created an urgent need for new solutions to such new infections (Zandian et al. 2021). Human viral pathogens transmitted by water can be associated with moderate to high health significance; these viral pathogens, based on the WHO, are adenovirus, astrovirus, hepatitis A and E, rotavirus, norovirus and other enteroviruses (La Rosa 2020). Studies have been confirmed that a number of family of coronavirus can be survived in water systems, and clarified that viral loads (depending to population infection rates) can be presented in untreated wastewater (Ahmed et al. 2020). As known COVID-19 is a respiratory disease, however, large amounts of SARS-CoV-2 RNA have been found in patients' feces (Wu et al. 2020), as well as in raw sewage (Fongaro et al. 2021; La Rosa et al. 2020), sewage sludge (Peccia et al. 2020) and surface water (Guerrero-Latorre et al. 2020; Prado et al. 2021). Detecting the viable SARS-CoV-2 in urine (Sun et al. 2020) and feces (de Oliveira et al. 2021; W. Wang et al. 2020a, b) of patients has led to increasing the concerns about the possibility of transmission of COVID-19 by the fecal–oral or fecal–nasal routes. The existence of viable form of this virus in water and wastewater may be associated with issues for providing public health and difficulties in implementation of pandemic control strategies (Vickers 2017); this situation can be exacerbated in developing countries that do not have adequate access to sanitation and safe water. So far, credible evidence of transmission of COVID-19 through contaminated water has been achieved (La Rosa et al. 2020). Nonetheless, the need for research about the resistance of SARS-CoV-2 on environmental matrices, e.g., surface water and wastewater, has been emphasized by the World Health Organization (Organization 2020). One of the important sectors to protect the human health during such current pandemic is water services (Shutler et al. 2020b). Finding the SARS-CoV-2 in fecal samples as in untreated wastewater can be indicative of the possibility of its fecal–oral transmission. This possibility has been resulted in worries toward the transmission of this coronavirus into the environment recently. These worries is rising when the untreated or inadequately treated wastewater is released to environment since it may rise the risk infection with SARS-CoV-2 in waters (Arslan 2020; Cahill and Morris 2020). The water which is used for human consumption should be treated by conventional methods and chlorine-based disinfection correctly should be used to provide a residual chlorine level higher than 0.5 mg/L in the distribution network; this can help to fight SARS-CoV-2 (Wang et al. 2020a, b).

The results of studies have shown that coronavirus is able to survive in human feces for 3 days and in raw water without chlorine and hospital wastewater for 2 days at a temperature of 2 °C, and if the wastewater is not treated, the virus can enter surface and groundwater, and through it, it can cause environmental pollution and disease in humans (Lodder and de Roda Husman 2020; Medema et al. 2020). Coronaviruses are important pathogens in humans and animals. Previous research has proved the presence and survival of a number of virus types in groundwater resources (24–21). Therefore, COVID-19 can cause pathogenicity if it enters the water through various ways such as cemetery sewage, hospital effluent and domestic sewage. In 2020, Naddeo et al. conducted a study on the fate of the coronavirus in the urban water cycle. The results showed that COVID-19 virus can easily enter surface water sources through sewage and cause disease in a large number of people (Dargahi et al. 2021a, b; Naddeo and Liu 2020).

Providing safe water to protect human health is essential during the spread of all infectious diseases, including coronavirus 2019 (COVID-19). In terms of the prevalence of coronavirus (SARS-CoV-2) in the world and its identification in municipal wastewater, the possibility of the presence of this virus in water sources and services is raised. Therefore, examining the presence of coronavirus (SARS-CoV-2) in water resources and services is a new topic and can be studied from different aspects and is considered important in terms of quality control of water resources and services. Identifying it in water sources and services can also be an important aid in detecting the virus in the environment. Therefore, this study aimed to identify coronavirus and determine physicochemical properties including pH, TDS, residual chlorine, turbidity and temperature in surface water (rivers, dams and lake), ground water (well water, springs), water distribution networks and water treatment plants (WTP) in Ardabil province.

## Materials and methods

**Fig. 1:**
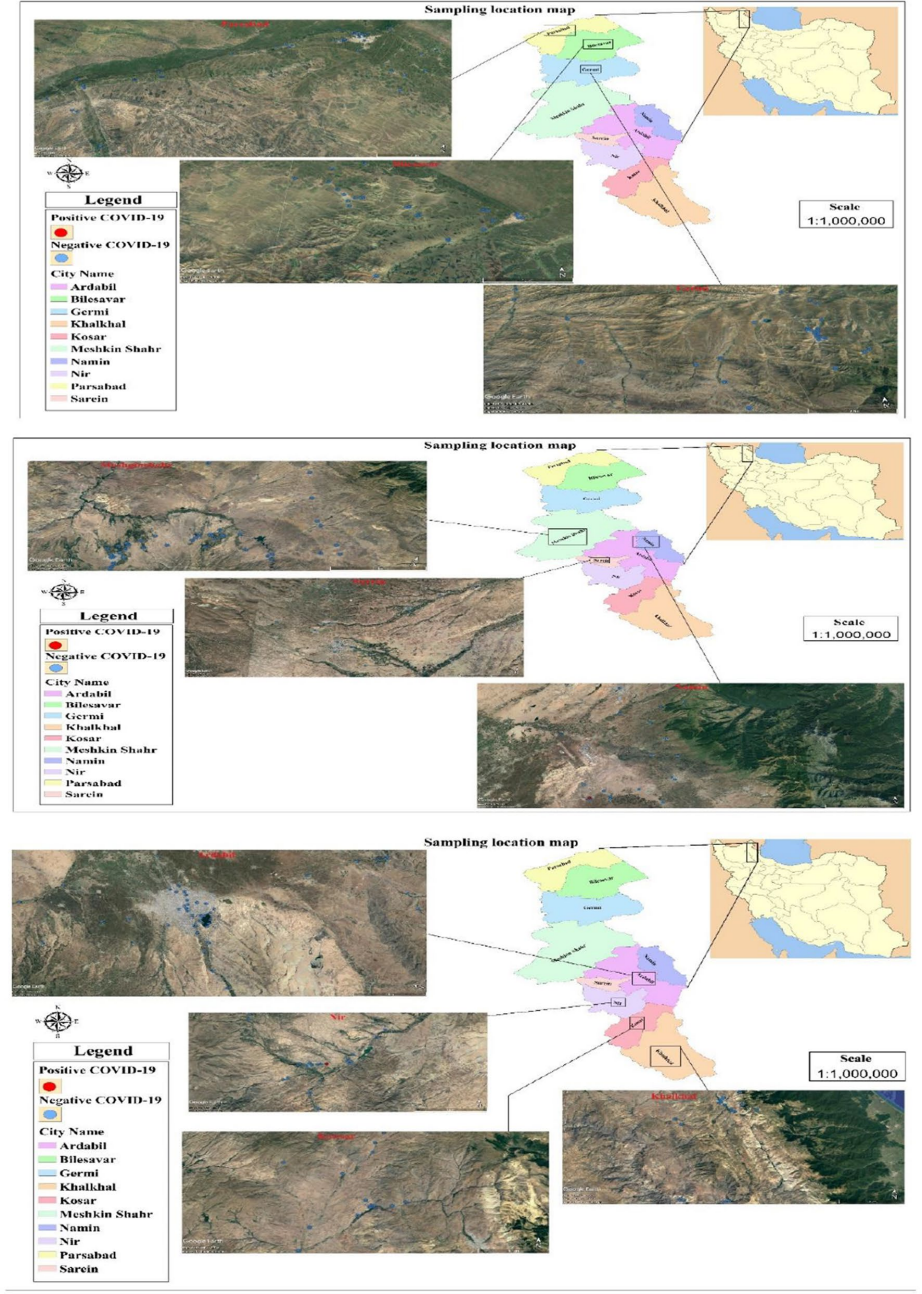
Position of different sampling points in this study

**Fig. 2:**
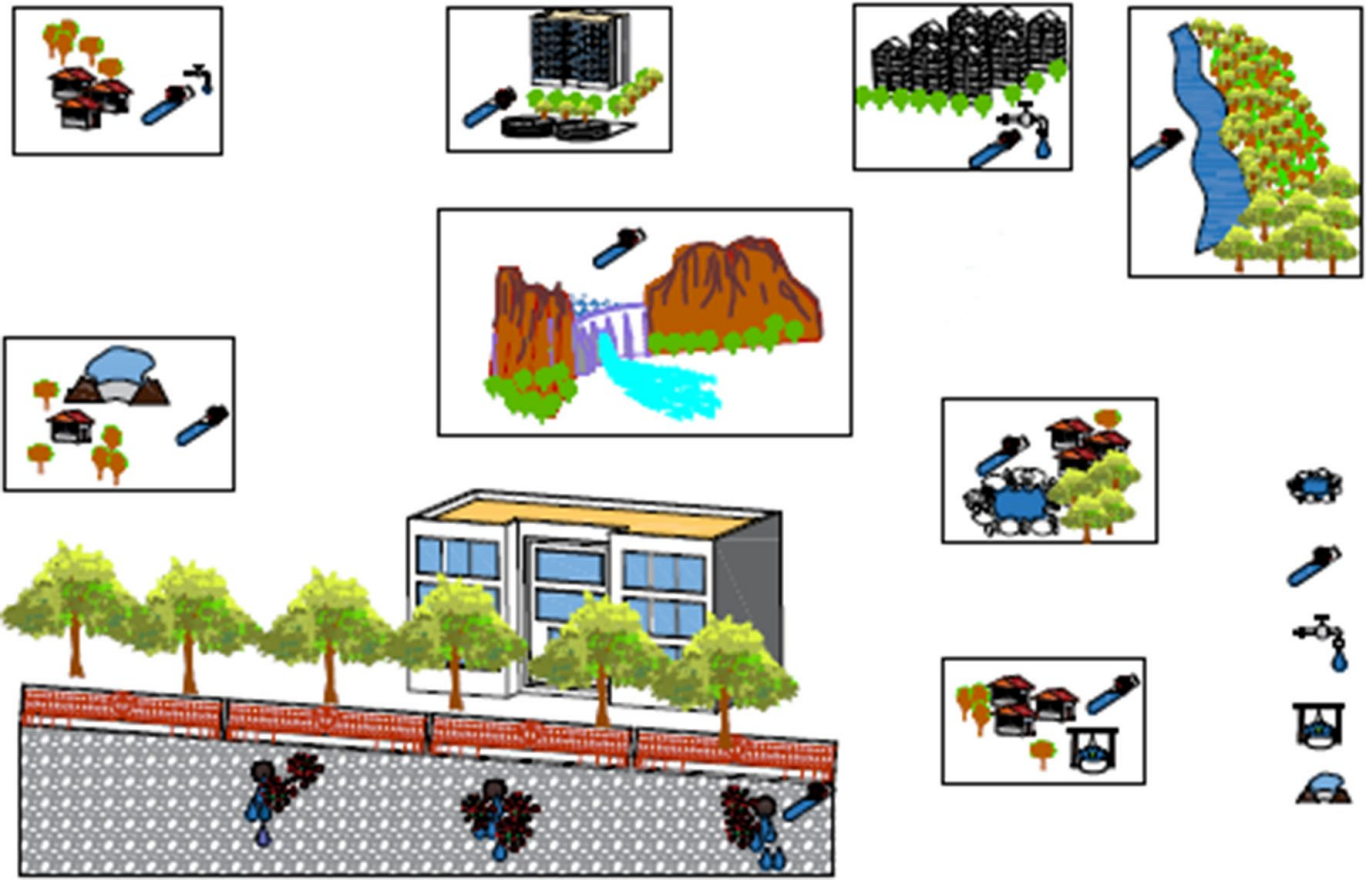
Schematic of SARS –CoV-2 transmission pathways in water sources

In this descriptive analytical study, the water resources and services of North West Iran were collected. Samples were selected for the prevalence of coronavirus (SARS-CoV-2) in the water resources and services of Ardabil province. Methodology was based on collecting available information from various sources (reports, articles and databases) to determine the main and important rivers, lakes and dams in Ardabil province. In the present study, water resources included water transmission lines, storage tanks, drinking water distribution network, groundwater resources as well as water treatment plants in Ardabil province. For this purpose, out of 6 sub-basins in Ardabil province, 2 sub-basins (Darreh-e-Rood and Balharud) related to Aras’s catchment and 2 sub-basins (Ghezel Ozan and Hiruchay) related to Ghezel Ozan catchment (4 basins in total), it was selected to cover most of the cities of the province. The rivers associated with the four basins identified in this study included: Balkhali Chai, Qarasu, Ghezel Ozan, Hiro Chai, Aras, Khiavchai, Balharud, Givi Chai and Garmi Chai, Mill Moghan, Givi, Gilarloo, Yamchi and Sabalan dams, and Ardabil, Bilesvar, Jafarabad, Roh Kennedy and Parsabad water treatment plants were selected examples of surface water resources. Transmission lines, water canals, storage tanks and drinking water distribution network of Ardabil cities of Ardabil province (Ardabil, Pars Abad, Garmi, Bilesvar, Aslandooz, Meshkinshahr, Nir, Namin, Khalkhal, Jafarabad, Ingut) and suburban villages in this study were considered. Also, wells and springs in the cities of Ardabil province were selected sources of groundwater. 5 L per sample was collected in special glass containers in the package. Samples will be collected using the combined sampling method (by sampling from a specific point at different times and combining them together). The samples will be transferred to the laboratory on ice and the samples will be stored at 4 °C until analysis. Finally, distribution and qualitative zoning map of surface water resources were done through GIS software (Fig. 1). In the present study, 267 samples from different water sources in northwest of Iran (Ardabil province) were examined to identify SARS-CoV-2 virus, and the results are presented in Table 1. In each sample of water collected, different parameters such as residual chlorine (VAHEB, VE611, DPD) pH (VAHEB, VE611) phenol red (VAHEB, VE611, DPD), turbidity (AQUA LVTiCAL250T-IR), total dissolved solids (TDS) (DMT-20) and temperature (DMT-20) were also measured. The physicochemical results of samples related to water sources of Ardabil province are presented in Table 1. Sampling was performed in August, September and February 2020 to March and April 2021. Filtered samples were prepared by consecutively filtering through with 0.45 μm and 0.22 and 0.1 μm pore size filters. The samples will then be centrifuged for 12 min at 12,000 rpm. The supernatant is discarded and extracted into the remaining micro tubes. Cutoff Ct value in several kits was used to identify SARS coronavirus-2 in water samples < 40. In addition, a schematic of coronavirus transmission pathways in water sources is presented in Fig. 2. In this figure, the possibility of transmitting coronavirus to water sources has been determined.

**Table 1:** Coronavirus (SARS-CoV-2) outbreak on water sources and treatment plants in northwest of Iran (*n* = 267).

Sample code	Sampling location	Type of Water Source	Residual chlorine (ppm)	pH	Turbidity (NTU)	TDS (mg/L)	Temperature (°C)	PdRp gene (Ct)	N gene (Ct)	Result
1	Khalkhal	River	0	8.2	22	201	5.2	ND	ND	Neg
2	Khalkhal	River	0	8.1	22.3	201.5	5.6	ND	ND	Neg
3	Khalkhal	Spring	0	8.7	0	391	7.1	ND	ND	Neg
4	Khalkhal	Spring	0	8.1	0	104	0.9	ND	ND	Neg
5	Khalkhal	Dam	0	7.2	0	233	1	ND	ND	Neg
6	Khalkhal	Spring	0	7.3	0	146	12.3	ND	ND	Neg
7	Khalkhal	Spring	0	7.3	0	307	10.5	ND	ND	Neg
8	Khalkhal	River	0	8	25.6	168	6.4	ND	ND	Neg
9	Khalkhal	River	0	8.1	41	243	6.2	ND	ND	Neg
10	Khalkhal	River	0	8	41	227	4.7	ND	ND	Neg
11	Khalkhal	River	0	8.2	48	262	6.4	ND	ND	Neg
12	Khalkhal	Spring	0	7.1	0	201	7.5	ND	ND	Neg
13	Kowsar	Dam	0	8	40.2	278	3.5	ND	ND	Neg
14	Kowsar	Spring	0	7.4	0	177	4.4	ND	ND	Neg
15	Ardabil	River	0	7.7	0	221	7.5	ND	ND	Neg
16	Ardabil	River	0	7.8	8	194	7.8	ND	ND	Neg
17	Ardabil	River	0	8	32	299	8.2	ND	ND	Neg
18	Namin	River	0	8.2	50	192	6.2	ND	ND	Neg
19	Namin	River	0	8.4	45	279	6.3	ND	ND	Neg
20	Namin	Spring	0	7.4	17.1	53	6.1	ND	ND	Neg
21	Namin	Water stream	0	7.6	20	182	5.1	ND	ND	Neg
22	Namin	River	0	8.2	57	9	3.2	ND	ND	Neg
23	Namin	Tap water	0	7.6	0	234	8.6	ND	ND	Neg
24	Namin	Tap water	0	7.7	0	190	11.1	ND	ND	Neg
25	Namin	Tap water	0.1	7.6	0	189	10.5	ND	ND	Neg
26	Namin	Tap water	0	7.9	0	282	7.2	ND	ND	Neg
27	Namin	Tap water	0.5	7.4	0	126	6.3	ND	ND	Neg
28	Ardabil	River	0	7.8	30	823	6.8	ND	ND	Neg
29	Ardabil	Tap water	0.6	7.6	0	333	7.1	ND	ND	Neg
30	Khalkhal	River	0	7.7	10.6	189	6.8	ND	ND	Neg
31	Khalkhal	River	0	7.7	10	176	7.1	ND	ND	Neg
32	Khalkhal	River	0	7.8	26	1827	6.4	ND	ND	Neg
33	Khalkhal	River	0	7.6	25.5	1749	6.2	ND	ND	Neg
34	Ardabil	Tap water	0	7.2	0	427	6.1	ND	ND	Neg
35	Nir	River	0	7.1	16	143	3.3	ND	ND	Neg
36	**Nir**	**River**	**0**	**6.4**	**60**	**172**	**3.2**	**34.2**	**31.34**	**Pos**
37	Nir	Tap water	0.1	6.9	0	100	4.8	ND	ND	Neg
38	Nir	River	0	7.8	11.6	133	7.4	ND	ND	Neg
39	Nir	River	0	7.4	10.8	138	7.2	ND	ND	Neg
40	Nir	River	0	7.2	24	713	4.1	ND	ND	Neg
41	Nir	River	0	7.1	21	441	4.5	ND	ND	Neg
42	Nir	Tap water	0	6.7	0	721	7.2	ND	ND	Neg
43	Nir	Tap water	0.1	7	0	113	9.1	ND	ND	Neg
44	Nir	Tap water	0.1	7.1	0	145	8.5	ND	ND	Neg
45	Nir	Tap water	0.1	7.2	0	105	6.6	ND	ND	Neg
46	Nir	River	0	8.5	15	127	6.5	ND	ND	Neg
47	Nir	Dam	0	8.4	26	381	6.3	ND	ND	Neg
48	Nir	Dam	0	8.5	25.6	381	5.5	ND	ND	Neg
49	Nir	Dam	0	8.5	29	381	6.1	ND	ND	Neg
50	Nir	Dam	0	8.2	26.1	384	7.3	ND	ND	Neg
51	Sarein	Tap water	0.2	7.8	0	165	7.5	ND	ND	Neg
52	Sarein	River	0	7.1	240	414	13.8	ND	ND	Neg
53	Sarein	Tap water	0.1	7.5	0	225	3.2	ND	ND	Neg
54	Sarein	Tap water	0.2	7.4	0	191	4.6	ND	ND	Neg
55	Sarein	River	0	7.6	14	114	5.2	ND	ND	Neg
56	Sarein	River	0	7.8	22	159	4.8	ND	ND	Neg
57	Sarein	Tap water	0	7.2	0	176	6.4	ND	ND	Neg
58	Sarein	Tap water	0	7.2	0	176	6.5	ND	ND	Neg
59	Sarein	Water well	0	7.8	0	380	10.3	ND	ND	Neg
60	Ardabil	Tap water	0.2	7.3	0	216	3.7	ND	ND	Neg
61	Ardabil	River	0	7.4	30	519	5.5	ND	ND	Neg
62	Ardabil	River	0	7.5	30.2	711	4.8	ND	ND	Neg
63	Meshginshahr	River	0	7.1	16.4	971	4.7	ND	ND	Neg
64	Meshginshahr	River	0	7.7	21.5	739	7.6	ND	ND	Neg
65	Meshginshahr	River	0	7.9	21	736	4.6	ND	ND	Neg
66	Meshginshahr	Dam	0	7.8	29	562	6.7	ND	ND	Neg
67	Meshginshahr	Tap water	0	7.2	0	384	6.2	ND	ND	Neg
68	Meshginshahr	Agricultural drainage	0	7.1	14	256	5.9	ND	ND	Neg
69	Meshginshahr	River	0	8.9	242	365	7.1	ND	ND	Neg
70	Meshginshahr	River	0	7.6	100	57	7.6	ND	ND	Neg
71	Meshginshahr	River	0	7.4	32	80	6.8	ND	ND	Neg
72	Meshginshahr	River	0	7.6	22.5	234	5.8	ND	ND	Neg
73	Meshginshahr	River	0	7.6	22	245	6.2	ND	ND	Neg
74	Meshginshahr	Tap water	0.8	7.4	0	106	5.6	ND	ND	Neg
75	Meshginshahr	Tap water	0.5	7.4	0	110	6.3	ND	ND	Neg
76	Meshginshahr	Tap water	0.4	7.3	0	112	6.8	ND	ND	Neg
77	Meshginshahr	Tap water	0	7.6	0	30	2.3	ND	ND	Neg
78	Meshginshahr	Tap water	0.2	7.2	0	49	5.8	ND	ND	Neg
79	Meshginshahr	River	0	7.4	27.1	223	3.8	ND	ND	Neg
80	Meshginshahr	River	0	7.4	11.5	223	3.9	ND	ND	Neg
81	Meshginshahr	Tap water	0	7.4	0	23	4.5	ND	ND	Neg
82	Meshginshahr	Tap water	0	7.2	0	130	5.6	ND	ND	Neg
83	Meshginshahr	River	0	8.4	13.8	305	2.5	ND	ND	Neg
84	Meshginshahr	Tap water	0.1	6.9	0	84	4.9	ND	ND	Neg
85	Meshginshahr	River	0	7.8	160	261	6.2	ND	ND	Neg
86	Meshginshahr	River	0	7.9	24	424	7.8	ND	ND	Neg
87	Meshginshahr	Spring	0	6.8	0	447	11.8	ND	ND	Neg
88	Meshginshahr	River	0	7.9	16.8	337	5.4	ND	ND	Neg
89	Meshginshahr	Water well	0	6.8	0	403	10.9	ND	ND	Neg
90	Meshginshahr	Tap water	0.4	6.9	0	692	9.1	ND	ND	Neg
91	Meshginshahr	Spring	0	7.4	0	309	10.3	ND	ND	Neg
92	Kowsar	Dam	0	8	28	367	7.2	ND	ND	Neg
93	Kowsar	River	0	8.2	14	290	4.9	ND	ND	Neg
94	Kowsar	River	0	8.2	28.6	249	5.8	ND	ND	Neg
95	Kowsar	River	0	8.2	12.1	349	5.1	ND	ND	Neg
96	Kowsar	River	0	8.6	32.4	261	4.7	ND	ND	Neg
97	Kowsar	Tap water	0.2	7.6	0	259	6.4	ND	ND	Neg
98	Kowsar	River	0	8	28	301	4.9	ND	ND	Neg
99	Germi	River	0	8.2	22.7	2490	6.1	ND	ND	Neg
100	Bilesavar	Tap water	0.2	7.6	0	356	5.5	ND	ND	Neg
101	Meshginshahr	Tap water	0	7.4	0	379	6.8	ND	ND	Neg
102	Ardabil	Tap water	0.2	7.9	0	245	12.1	ND	ND	Neg
103	Ardabil	Tap water	0.3	7.3	0	263	8.4	ND	ND	Neg
104	Ardabil	Tap water	0.1	7.6	0	310	7.6	ND	ND	Neg
105	Ardabil	Tap water	0.1	7.5	0	384	14.1	ND	ND	Neg
106	Ardabil	Tap water	0.5	7.8	0	412	9.8	ND	ND	Neg
107	Ardabil	Shorabil Lake	0	7.6	11.6	1259	6.5	ND	ND	Neg
108	Ardabil	Shorabil Lake	0	7.6	11.3	1709	4.9	ND	ND	Neg
109	Ardabil	Shorabil Lake	0	7.6	11.2	845	8.5	ND	ND	Neg
110	Ardabil	Shorabil Lake	0	7.6	11.4	848	6.2	ND	ND	Neg
111	Ardabil	River	0	9.8	10.1	815	7.9	ND	ND	Neg
112	Ardabil	Shorabil Lake	0	10.2	10.4	855	7.6	ND	ND	Neg
113	Ardabil	Shorabil Lake	0	10.2	10.8	858	7.8	ND	ND	Neg
114	Ardabil	Tap water	0.3	7.6	0	350	10.7	ND	ND	Neg
115	Ardabil	Tap water	0.3	7.6	0	349	10.5	ND	ND	Neg
116	Ardabil	River	0	10.2	12.8	772	8.4	ND	ND	Neg
117	Ardabil	River	0	10.2	11.9	769	9.4	ND	ND	Neg
118	Ardabil	River	0	10	7.8	747	10.1	ND	ND	Neg
119	Ardabil	River	0	10.2	8.1	693	10.5	ND	ND	Neg
120	Ardabil	Tap water	0	9	0	377	10.1	ND	ND	Neg
121	Ardabil	Tap water	0.3	7.8	0	383	9.8	ND	ND	Neg
122	Ardabil	Water pool	0	11	10	486	9.5	ND	ND	Neg
123	Ardabil	Tap water	0.4	7.6	0	322	11.1	ND	ND	Neg
124	Ardabil	River	0	10	8.6	726	9.4	ND	ND	Neg
125	Ardabil	Tap water	0.3	7.6	0	345	6.7	ND	ND	Neg
126	Ardabil	River	0	9.8	8.7	810	8.8	ND	ND	Neg
127	Ardabil	River	0	10	9.2	800	9.3	ND	ND	Neg
128	Ardabil	River	0	10	9.8	757	10.6	ND	ND	Neg
129	Ardabil	Tap water	0	7.8	7.6	311	12.5	ND	ND	Neg
130	Kowsar	River	0	8.2	12.3	356	8.3	ND	ND	Neg
131	Meshginshahr	River	0	7.8	10.4	539	13.5	ND	ND	Neg
132	Meshginshahr	River	0	8.4	10.1	700	12.1	ND	ND	Neg
133	Meshginshahr	River	0	8.4	11.2	419	12.4	ND	ND	Neg
134	Meshginshahr	River	0	7.6	10	302	12.7	ND	ND	Neg
135	Meshginshahr	Spring	0	8	6.5	483	6.1	ND	ND	Neg
136	Germi	River	0	8.4	36.1	253	15.9	ND	ND	Neg
137	Germi	River	0	8.2	9.5	377	18.1	ND	ND	Neg
138	Germi	Tap water	0	7.6	0	398	17.2	ND	ND	Neg
139	Germi	Tap water	0.1	7.4	0	358	8.2	ND	ND	Neg
140	Germi	Dam	0	10.2	26.7	1394	19.8	ND	ND	Neg
141	Germi	Tap water	0.5	7.6	0	483	8.6	ND	ND	Neg
142	Germi	Tap water	0.4	7.6	0	489	7.2	ND	ND	Neg
143	Germi	Tap water	0.4	7.5	0	381	6.8	ND	ND	Neg
144	Germi	River	0	7.2	45.2	967	20.2	ND	ND	Neg
145	Germi	River	0	7.2	44.6	1039	19.1	ND	ND	Neg
146	Germi	Tap water	0.2	7.4	0	490	17.9	ND	ND	Neg
147	Germi	River	0	7.4	22.1	845	18.2	ND	ND	Neg
148	Germi	River	0	7.4	18.9	863	18.6	ND	ND	Neg
149	Germi	River	0	7.4	18.5	971	20.2	ND	ND	Neg
150	Germi	River	0	7.4	18.1	967	20.5	ND	ND	Neg
151	Germi	River	0	7.6	26.5	683	23.8	ND	ND	Neg
152	Bilesavar	Tap water	0	7.4	0	412	11.6	ND	ND	Neg
153	Germi	Tap water	0.3	7.4	0	352	12.2	ND	ND	Neg
154	Germi	Tap water	0.3	7.4	0	361	11.8	ND	ND	Neg
155	Bilesavar	River	0	9	36.2	2700	23.1	ND	ND	Neg
156	Bilesavar	Tap water	0.5	7.6	0	478	19.4	ND	ND	Neg
157	Bilesavar	Tap water	0.3	7.6	0	381	20.1	ND	ND	Neg
158	Bilesavar	Water Channel	0	7.9	41.3	487	19.7	ND	ND	Neg
159	Bilesavar	Water Channel	0	7.4	9.8	480	18.2	ND	ND	Neg
160	Bilesavar	Water Channel	0	7.4	10	428	19.4	ND	ND	Neg
161	Parsabad	Water Channel	0	7.4	10.5	480	18.5	ND	ND	Neg
162	Parsabad	River	0	8.2	42.1	1853	21.6	ND	ND	Neg
163	Parsabad	River	0	8.2	40.2	1840	21.2	ND	ND	Neg
164	Parsabad	Water Channel	0	8.6	11.2	478	17.6	ND	ND	Neg
165	Parsabad	Water Channel	0	8.6	10.8	473	17.1	ND	ND	Neg
166	Parsabad	Water Channel	0	8	10.5	473	17.3	ND	ND	Neg
167	Parsabad	Tap water	0.3	7.6	0	389	17.5	ND	ND	Neg
168	Parsabad	Tap water	0.4	7.6	0	392	16.8	ND	ND	Neg
169	Parsabad	Tap water	0.3	7.6	0	407	16.4	ND	ND	Neg
170	Parsabad	Water well	0	7.6	0	2596	20.5	ND	ND	Neg
171	Parsabad	River	0	8.6	36.2	523	21.1	ND	ND	Neg
172	Parsabad	River	0	7.6	10.9	481	17.2	ND	ND	Neg
173	Parsabad	River	0	10.1	15.5	848	18.6	ND	ND	Neg
174	Parsabad	River	0	10.5	15.2	853	19.2	ND	ND	Neg
175	Parsabad	River	0	10.3	15.8	865	19.3	ND	ND	Neg
176	Parsabad	River	0	10.6	16.1	856	19.5	ND	ND	Neg
177	Parsabad	River	0	10.9	15.4	844	19.8	ND	ND	Neg
178	Parsabad	River	0	10.1	14.9	857	18.5	ND	ND	Neg
179	Parsabad	River	0	9.2	28.1	1433	19.1	ND	ND	Neg
180	Parsabad	River	0	9	26.2	1439	19.5	ND	ND	Neg
181	Parsabad	Water well	0	7.8	0	481	23.8	ND	ND	Neg
182	Parsabad	Tap water	0	7.4	0	401	16.7	ND	ND	Neg
183	Bilesavar	WTP influent ^(1)^	0	7.8	28.6	582	8.2	ND	ND	Neg
184	Bilesavar	WTP effluent ^(2)^	1.5	7.8	0	474	9.5	ND	ND	Neg
185	Bilesavar	WTP influent ^(3)^	0	7.6	30.7	420	8.4	ND	ND	Neg
186	Bilesavar	WTP effluent ^(4)^	1.0	7.6	0	410	8.6	ND	ND	Neg
187	Bilesavar	Tap water	0.4	7.6	0	523	7.6	ND	ND	Neg
188	Bilesavar	Tap water	0.4	7.7	0	520	8.5	ND	ND	Neg
189	Bilesavar	River	0	9	11.5	510	9.4	ND	ND	Neg
190	Bilesavar	WTP influent ^(5)^	0	7.8	26.1	566	10.2	ND	ND	Neg
191	Bilesavar	WTP effluent ^(6)^	0.3	7.8	0	543	10.6	ND	ND	Neg
192	Bilesavar	Tap water	0.2	7.8	0	573	6.8	ND	ND	Neg
193	Bilesavar	River	0	7.8	10.3	582	7.9	ND	ND	Neg
194	Bilesavar	WTP influent ^(7)^	0	7.8	24.8	594	9.8	ND	ND	Neg
195	Bilesavar	WTP effluent ^(8)^	0.3	7.8	0	541	8.4	ND	ND	Neg
196	Bilesavar	River	0	7.8	10.6	584	10.8	ND	ND	Neg
197	Ardabil	Tap water	0	7.5	0	165	8.9	ND	ND	Neg
198	Ardabil	Tap water	0	7.7	0	178	8.4	ND	ND	Neg
199	Ardabil	Tap water	0	7.7	0	185	7.8	ND	ND	Neg
200	Ardabil	Tap water	0	7.4	0	166	7.5	ND	ND	Neg
201	Ardabil	Tap water	0	7.5	0	195	8.5	ND	ND	Neg
202	Ardabil	Tap water	0	7.4	0	174	9.1	ND	ND	Neg
203	Ardabil	Tap water	0	7.5	0	168	8.6	ND	ND	Neg
204	Ardabil	Tap water	0.5	7.4	0	312	7.6	ND	ND	Neg
205	Kowsar	Tap water	0.4	7.6	0	260	7.5	ND	ND	Neg
206	Kowsar	Tap water	0	7.6	0	251	7.1	ND	ND	Neg
207	Kowsar	Tap water	0	7.6	0	258	8.2	ND	ND	Neg
208	Kowsar	Tap water	0	7.6	0	266	8.6	ND	ND	Neg
209	Sarein	Tap water	0.4	7.3	0	182	6.7	ND	ND	Neg
210	Sarein	Tap water	0	6.8	0	165	7.5	ND	ND	Neg
211	Sarein	Tap water	0	6.8	0	168	7.9	ND	ND	Neg
212	Sarein	Tap water	0.2	6.8	0	192	6.8	ND	ND	Neg
213	Nir	Tap water	0	7	0	714	7.4	ND	ND	Neg
214	Nir	Tap water	0	7	0	554	6.8	ND	ND	Neg
215	Nir	Tap water	0	7	0	568	7.5	ND	ND	Neg
216	Nir	Tap water	0.3	6.8	0	105	7.2	ND	ND	Neg
217	Bilesavar	Tap water	0.1	7.8	0	125	10.6	ND	ND	Neg
218	Bilesavar	Tap water	0.5	7.8	0	135	11.2	ND	ND	Neg
219	Bilesavar	Tap water	0.1	7.8	0	145	9.5	ND	ND	Neg
220	Bilesavar	Tap water	0.1	7.8	0	138	9.2	ND	ND	Neg
221	Germi	Tap water	0.5	8.2	0	485	8.6	ND	ND	Neg
222	Germi	Tap water	0	7.6	0	455	8.4	ND	ND	Neg
223	Germi	Tap water	0	7.6	0	388	7.9	ND	ND	Neg
224	Germi	Tap water	0	7.6	0	375	7.5	ND	ND	Neg
225	Germi	Tap water	0	7.6	0	448	8.6	ND	ND	Neg
226	Germi	Tap water	0	7.6	0	396	8.5	ND	ND	Neg
227	Germi	Tap water	0	7.6	0	423	8.7	ND	ND	Neg
228	Namin	Tap water	0.8	7.8	0	192	9.1	ND	ND	Neg
229	Namin	Tap water	0	7.4	0	184	8.5	ND	ND	Neg
230	Namin	Tap water	0	7.2	0	174	8.8	ND	ND	Neg
231	Namin	Tap water	0.4	7	0	175	8.1	ND	ND	Neg
232	**Namin**	**Tap water**	**0.5**	7.4	0	126	6.5	**35.67**	**34.96**	**Pos**
233	Namin	Tap water	0.7	7.4	0	182	8.2	ND	ND	Neg
234	Namin	Tap water	0	7.2	0	186	8.8	ND	ND	Neg
235	Parsabad	Tap water	0	7.6	0	325	9.6	ND	ND	Neg
236	Parsabad	Tap water	0	7.3	0	314	9.2	ND	ND	Neg
237	Parsabad	Tap water	0.4	7.5	0	365	9.6	ND	ND	Neg
238	Parsabad	Tap water	0	6.8	0	389	8.9	ND	ND	Neg
239	Parsabad	Tap water	0	7.9	0	414	7.9	ND	ND	Neg
240	Parsabad	Tap water	0	7.7	0	426	8.5	ND	ND	Neg
241	Parsabad	Tap water	0	7.5	0	364	9.2	ND	ND	Neg
242	Parsabad	Tap water	0	7.6	0	386	9.5	ND	ND	Neg
243	Parsabad	Tap water	0.6	7.8	0	392	9.8	ND	ND	Neg
244	Meshginshahr	Tap water	0.6	7.2	0	114	6.5	ND	ND	Neg
245	Meshginshahr	Tap water	0	7.5	0	316	7.9	ND	ND	Neg
246	Meshginshahr	Tap water	0	7.1	0	286	7.3	ND	ND	Neg
247	Meshginshahr	Tap water	0	7.3	0	236	8.2	ND	ND	Neg
248	Meshginshahr	Tap water	0	7.1	0	292	6.9	ND	ND	Neg
249	Meshginshahr	Tap water	0.8	7.4	0	318	8.2	ND	ND	Neg
250	Meshginshahr	Tap water	0	7.2	0	312	8.5	ND	ND	Neg
251	Namin	Tap water	0.2	7.7	0	132	7.2	ND	ND	Neg
252	Namin	Tap water	0.2	7.5	0	145	8.4	ND	ND	Neg
253	Namin	Tap water	0	7.4	0	164	6.8	ND	ND	Neg
254	Parsabad	Tap water	0.3	7.9	0	314	9.7	ND	ND	Neg
255	Parsabad	Tap water	0.2	7.8	0	318	9.6	ND	ND	Neg
256	Parsabad	River	0	10.5	14.5	835	18.2	ND	ND	Neg
257	Parsabad	River	0	10.8	14.1	837	18.5	ND	ND	Neg
258	Parsabad	River	0	11.1	15.2	845	18	ND	ND	Neg
259	Parsabad	River	0	7.2	24.6	865	14.2	ND	ND	Neg
260	Germi	River	0	7.5	24.2	852	14	ND	ND	Neg
261	Germi	River	0	7.2	22	836	14	ND	ND	Neg
262	Germi	Tap water	0.2	7.6	0	326	10.2	ND	ND	Neg
263	Germi	Tap water	0	7.4	0	284	6.4	ND	ND	Neg
264	Germi	Tap water	0.3	7.2	0	364	9.8	ND	ND	Neg
265	Meshginshahr	River	0	8.1	12	485	16.5	ND	ND	Neg
266	Parsabad	WTP influent ^(9)^	0	7.1	5.5	480	9.5	ND	ND	Neg
267	Parsabad	WTP effluent ^(10)^	0.4	7.5	0	345	10.1	ND	ND	Neg

Bold means a positive coronavirus test in water sources

Influent of Bilesvar drinking water treatment plant (WTP); (2) Effluent of Bilesvar drinking WTP; (3) Influent of Qara Qasemlu village drinking WTP; (4) Effluent of Qara Qasemlu village drinking WTP; (5) Influent of Jafarabad drinking WTP; (6) Effluent of Jafarabad drinking WTP; (7) Influent of Rohkandi village drinking WTP; (8) Effluent of Rohkandi village drinking WTP; (9) Influent of Parsabad drinking WTP; (10) Effluent of Parsabad drinking WTP; *ND* Not detected; *Neg* Negative result; *Pos* Positive result.

### RNA extraction

Viral RNA was extracted from sample material and collected in elution buffer, using the High Pure Viral Nucleic Acid Kit at 200 µl. PCR amplification was performed using the SuperScript™ III One-Step RT-PCR System with Platinum™ Taq DNA Polymerase (Invitrogen, USA).

### RNA amplification

RNA segments of SARS-CoV-2 were amplified using two sets of primers (N and ORF1a/b) in each amplification reaction. These primer sets have been used as a rotini for the diagnosis of COVID-19 in human samples of Iran. The sequences of both the forward and reverse primers and probes used in this study are listed below: probe RdRP_SARSr-P2 F-ACAGGTGGAACCTCATCAGGAGATGC-BBQ, ORF1a/b SARSr-F GTGARATGGTCATGTGTGGCGG, ORF1a/b SARSr-R CARATGTT AAASACACTATTAGCATA, SARS-CoV-2 N gene primer and Probe, F-primer AAATTTTGGGGACC AGGAAC, R-primer TGGCAGCTGTGTAG GTCAA and probe PFAM-ATGTCGCGCATTGG CATGGA-BHQ. Cycling parameters 94 °C 3′ 94 °C 15″ 55 °C 10′ 58 °C 30″ 45x. suitable amount of the synthesis reagents for real-time PCR Master Mix: H2O (RNAse free) 0.6 μL 2 × reaction mix * 12.5 μL MgSO4 (50 mM) 0.4 μL BSA (1 mg/mL)** 1 μL primer ORF1a/b-F (10 μM stock solution), 1.5 μL primer ORF1a/b R (10 μM stock solution), 2 μL probe RdRP P1 (10 μM), 0.5 μL probe RdRP_SARSr-P2 (10 μM), 0.5 μL of SARS-CoV-2 primer and probe SARS-Cov-2 RdRP and N genes\Taq EnzymeMix* 1 μL template RNA, add 5 μL, total reaction mix 20 μL. \SARS-CoV-2 Wuhan type used as positive control in amplification assays of SARS-CoV-2 genetic material (González-González et al. 2021). The estimated lower limit of detection was ~ 1 copy of the N gene of SARS-CoV-2 per ml of water. The lowest positive value was 2.5 copies/ml.

### Data analysis method

After collecting the samples and analyzing them by PCR, the data obtained by EXEL and SPSS software version 20 were analyzed with similar articles and existing guidelines.

## Results and discussion

In the present study, after collecting 267 samples from different water sources in northwest of Iran (Ardabil province), they were tested to detect COVID-19 virus, the results of which are presented in Table 1. For the collected samples, measurement of the amount of parameters, e.g., residual chlorine, pH, turbidity, total dissolved solids (TDS) and temperature, was also taken. The results related to physicochemical characteristics of samples taken from water sources of Ardabil province are shown in Table 1. Sampling was taken in two months of 2020 (August and September) and three months of 2021 (February, March and April). In the present study, two samples were obtained to be “positive” and three replicate were considered for them. Out of 267 samples, two samples were detected to be positive, which their Ct values were 34.2 and 35.67. In the present study, the samples were collected from known depth (30–50 cm) of rivers, dams and lakes. Also, in Fig. 1, the general zoning map for the presence of COVID-19 virus has been shown.

Several studies have been performed in this area and have confirmed the presence of coronavirus in aquatic sources, while some studies have not been able to find coronavirus in aquatic sources. The presence and resistance of the coronavirus in the environment are a very important issue for researchers. The presence of coronavirus in human feces and sewage has been proved (Langone et al. 2020; Mancuso et al. 2021). Sewage is generated in corona treatment centers or residential areas where infected people live. One of the important points in detecting the coronavirus in water sources is the amount and method of collecting the virgin virus. The results of this study showed that there were two positive samples in water sampling that according to Table 1, both samples were taken from rivers in different cities. In studies that collect genetic material, it is more difficult to detect them (Rimoldi et al. 2020; Shutler et al. 2020a). Unlike bacteria, it is difficult to detect viruses due to the lack of cell culture to detect them. In the present study, the lack of detection of more positive samples may be due to the above. The reasons that can cause the virus undetectable in the water depends on various factors that cause the virus to eliminate. Among the effective factors in the elimination of viruses in water are the process of absorption, adhesion, sedimentation, inactivation due to temperature, sunlight, pH changes, salts and minerals (Giacobbo et al. 2021; Tran et al. 2020). On the other hand, viruses in water and wastewater come in contact with a variety of substances, including drugs, chemicals and detergents, which make the virus less detectable than it actually is (Kumar et al. 2021). Obtaining information on viral contamination of water and sewage can help diagnose the spread of the disease. In many cases, infected people have no symptoms but can be a source of virus production and excretion (Shutler et al. 2020a). Therefore, this information can be used as an epidemiological identification of the disease. On the other hand, analyzing the obtained data and also finding the relationship between the concentration and frequency of the virus in water and wastewater in different areas can help to predict critical and sensitive points (Adelodun et al. 2021).

The coronavirus is present in human feces, but its amounts are unknown (Bilal et al. 2020). If the virus enters surface water through household wastewater disposal, it can contaminate surface water (Bivins et al. 2020). If contaminated surface water is used for drinking, there is a risk of infection. This risk becomes significant when the contaminated water is not treated. Contaminated water can also enter water reservoirs and contaminate them on a large scale (Shutler et al. 2020a). In many developing cities, water treatment does not take place or water treatment is done in a basic way, and there is still the possibility of contamination. The World Health Organization recommends an effective dose of 5 mg/L to kill viruses in water. According to Iranian standard, the amount of residual chlorine for disinfection of water is between 0.2 and 0.8 mg per liter. In some places, according to the values mentioned, viruses may survive despite chlorination. Therefore, it is recommended to inspect different areas for chlorination in these conditions (Tran et al. 2020). If wastewater is disinfected in places where there are infected people (such as hospitals), many viruses survive with covering with other materials and the effect of chlorine on them is somewhat ineffective (Giacobbo et al. 2021; Tran et al. 2020). In this study, the main parameters of water quality were also tested. In the present study, the measured parameters were turbidity, pH, total dissolved solid (TDS) and temperature along with the contamination of water with coronavirus that mean values of these parameters are shown in Table 2. The relationship between the various parameters is shown in Table 3. Environmental factors may affect the survival of viruses in the aquatic environment. Due to the small number of positive samples in the present study, it was not possible to establish a statistical relationship between positive cases and environmental parameters. However, the values of positive environmental samples in the river for residual chlorine, pH, turbidity, total dissolved solid and temperature were 0 mg/L, 6.4, 60 NTU, 172 mg/L and 3.2 °C, respectively. Also, the environmental parameters of the positive samples in the tap for residual chlorine, pH, turbidity, TDS and temperature were 0.5 mg/L, 7.4, 0 NTU, 126 mg/L and 6.5 °C, respectively. Due to the small number of positive samples in our study, the effect of environmental factors is ambiguous and more studies are needed. On the other hand, some limited studies have been done in the environment. According to the obtained statistical test results, there was a significant relationship between pH and other water quality parameters studied in this study. TDS also had a standard relationship with other environmental parameters. Chlorine also had a statistically significant relationship with other environmental parameters. But no significant relationship was found between temperature and turbidity. Most studies have confirmed the relationship between residual chlorine and pH (Yang and Cheng 2007). Given that chlorine may be the most important factor in killing viruses in the aquatic environment, controlling pH and chlorine helps control viruses and other microorganisms in the aquatic environment, because the amount of residual chlorine is dependent on BPH, so that at lower pHs, its disinfection power increases.

**Table 2:** Mean values of environmental parameters

Temperature	9.6127	4.78927	267
pH	7.8536	.86112	267
TDS	454.2678	392.67634	267
Turbidity	12.3655	26.08768	267
Cl	.0963	.19996	267

**Table 3:** Statistical relationship between environmental parameters studied in this study

		Temperature	pH	TDS	Turbidity	Cl
Temperature	Pearson Correlation	1	.307**	.460**	.049	− .053
	Sig. (two-tailed)		.000	.000	.424	.390
	N	267	267	267	267	267
pH	Pearson Correlation	.307^**^	1	.350^**^	.132^*^	− .178^**^
	Sig. (two-tailed)	.000		.000	.031	.004
	N	267	267	267	267	267
TDS	Pearson Correlation	.460**	.350**	1	.129*	− .172**
	Sig. (two-tailed)	.000	.000		.036	.005
	N	267	267	267	267	267
Turbidity	Pearson Correlation	.049	.132*	.129*	1	− .229**
	Sig. (two-tailed)	.424	.031	.036		.000
	N	267	267	267	267	267
Cl	Pearson Correlation	− .053	− .178**	− .172**	− .229**	1
	Sig. (two-tailed)	.390	.004	.005	.000	
	N	267	267	267	267	267

**Correlation is significant at the 0.01 level (two-tailed)

*Correlation is significant at the 0.05 level (two-tailed)

A study by (Adelodun et al. 2021) showed that SARS-CoV-2 virus can be found in the environment with varying degrees of resistance and survival. In their review article, they did not definitively confirm the transmission of the virus through food, water and other environmental components. In the present study, the persistence and status of viruses in water have not been studied. In some cases mentioned in Table 1, positive samples have been found. de Oliveira et al. in their study pointed to water and sewage contamination with the coronavirus, which may cause concerns through fecal–oral transmission (de Oliveira et al. 2021). However, this study noted that fecal–oral transmission was not established. Although the transmission of the virus from water is ambiguous, but considering that in our study in the two cities of the province, the sample of coronavirus has become positive, it is better to take serious health recommendations. Believe that in areas where sanitation is not done properly, viruses can reach the main body of water resources and survive for a long time. Their study also showed that low seasonal temperatures increase the risk of transmitting the virus through water. But the survival of viruses depends on the environment.

Our study was conducted at ambient temperature, because our samples were taken from the environment. Studies have been performed on the survival of viruses at different temperature changes. Bivins et al. (2020) reported that the SARS-CoV-2 virus survives in water and wastewater for 1.5 and 1.7 days, respectively. At 50 and 70 °C, T_90_ (the time for 90% reduction) decreased relative to ambient temperature. In fact, as temperatures rise, viruses’ resistance decreases. In our previous study, we investigated SARS-CoV-2 in municipal wastewater treatment plant, collection network and hospital wastewater that found some positive samples in effluent of wastewater treatment (Dargahi, et al. 2021a, b). Kumar et al. (2021) reported that viruses lose their infectious ability to deal with wastewater that may contain alcohol-based detergents. In some areas where rainwater mixes with municipal wastewater, it may also become contaminated, although it will help dilute the wastewater. Tran et al. (2020) have stated that in addition to viral contamination of water sources by mixing them with sewage, contaminated masks of patients left in the environment can also be another cause of contamination of water sources with coronavirus. The results of their study showed that the survival of coronavirus in water sources is strongly related to temperature, water properties and concentration of suspended solids, pH and concentration of disinfectants. The World Health Organization considers conventional water purification and chlorination processes sufficient to kill viruses and bacteria. de Oliveira et al. (2021) also considered the influence of environmental factors to be effective. Their study found that temperature had a significant effect on the resistance and survival of the virus in the aquatic environment. They showed T_90_ levels of 7.7 and 5.5 for river water and wastewater at 4 °C, respectively. But with increasing temperature, this resistance has decreased. Rimoldi et al. (2020) reported that they found SARS-CoV-2 viral RNAs in water sources. However, the tests for infectivity in the laboratory were negative. This indicates a low risk of water pollution caused by SARS-CoV-2. Mancuso et al. stated that since most fresh water is used for agriculture, the quality of water for irrigation should be ensured. Coronaviruses can infect agricultural products and cause contamination via irrigation (Mancuso et al. 2021). Different results have been reported in the studies. In a study by Mahlknecht et al. (2021) that carried out at the height of the corona pandemic took samples from the water sources of Monterrey. Their results were analyzed by PCR which revealed that 44% of sources near the city were positive with a virus load of 2.6 to 38.3 Copies/ml. Also, 12% of the samples taken from the dams and 13% of the samples taken from the rivers were positive. A total of 50% of their samples were positive for coronavirus. Our study showed that 2 cases of the all taken samples were positive regarding the presence of SARS-CoV-2. Positive samples were taken from river and tab.

## Conclusions

The existence of viable form of SARS-CoV-2 in water is associated with the public health measures. Among the effective factors in the elimination of viruses in water are the process of absorption, adhesion, sedimentation, inactivation due to temperature, sunlight, pH changes, salts and minerals. Chlorine and pH, which were significantly associated in this study, are one of the factors that can control the coronavirus in aquatic environments. Difficulties in implementation of pandemic control strategies can be exacerbated in developing countries that do not have adequate access to sanitation and safe water. The results of this study showed that there were two positive samples in water sampling that according to, positive samples were taken from rivers and tab in different cities. Although the transmission of the virus from water is ambiguous, but considering that in our study in the two cities of the province, the sample of coronavirus has become positive, it is better to take serious health recommendations. Environmental health engineering believes that in areas where sanitation is not done properly and viruses can reach the main body of water resources and survive for a long time.

## Data Availability

The dataset analyzed during the current study is available from the corresponding authors on realistic demand.
